# Cue-Restricted Smoking as a behavioral adjunct for smoking cessation: Observational sub-analysis of a randomized trial of deep transcranial magnetic stimulation

**DOI:** 10.18332/tpc/209189

**Published:** 2025-10-03

**Authors:** Jaqueline R. Scholz, Bianca B. Bellini, Sara D. V. Ziotti, Tania O. Abe, Debora Arnaut, Rodrigo L. Alberto, Marco A. Marcolin, Serena Tonstad

**Affiliations:** 1 Department of Prevention and Rehabilitation, Tobacco Treatment, Instituto do Coração, Hospital das Clínicas, Faculdade de Medicina, Universidade de São Paulo, São Paulo, Brazil; 2 Departamento de Neurologia, Hospital das Clinicas HCFMUSP, Faculdade de Medicina, Universidade de Sao Paulo, Sao Paulo, Brazil; 3 Cardiovascular Prevention Department, Oslo University Hospital, Oslo, Norway

**Keywords:** smoking cessation, transcranial magnetic stimulation, nicotine dependence, behavioral technique, Cue-Restricted Smoking

## Abstract

**INTRODUCTION:**

Cue-Restricted Smoking (CRS) is a behavioral technique for smoking cessation that has shown efficacy as an adjunct to pharmacotherapy. In CRS, individuals limit smoking to a standing position while facing a wall in an isolated environment devoid of sensory stimulation. This study aimed to assess the potential impact of CRS in a randomized trial evaluating deep transcranial magnetic stimulation (dTMS), which failed to show significant treatment effects.

**METHODS:**

In a randomized, double-blind, sham-controlled trial evaluating dTMS for smoking cessation, 100 participants were instructed to quit smoking by the end of week 1. None achieved cessation. Only 85 participants remained in the protocol beyond week 1 and received guidance to implement CRS during cravings. Cigarette consumption was monitored through self-report, exhaled carbon monoxide (COex), and plasma cotinine levels. Participants were categorized as: no reduction, additional reduction, or cessation. Joinpoint regression was used to assess longitudinal trends.

**RESULTS:**

The 85 participants (68% male; mean age 49 ± 11.6 years) reported a mean of 31 ± 11.2 years of smoking. Beyond week one, 33% had not reduced their consumption, 8% showed a mild reduction, 43% a moderate reduction, and 15% a significant reduction; none achieved cessation. After CRS, 40% (95% CI: 29.5–51.2) reported further reduction, and 16.5% (95% CI: 9.3–26.1) achieved verified cessation by week 12. Joinpoint analysis confirmed significant decreasing trends in cigarette use and COex among the significant reduction and cessation groups.

**CONCLUSIONS:**

CRS was associated with clinically meaningful reductions in smoking and biochemically confirmed cessation. It is a low-cost, scalable technique that does not require intensive training or pharmacotherapy. This approach may be advantageous in low-resource settings. It warrants evaluation in larger randomized trials across diverse populations.

**CLINICAL TRIAL REGISTRATION:**

The study is registered on the official website of ClinicalTrials.gov

**IDENTIFIER:**

ID NCT03264313

## INTRODUCTION

The gold standard for tobacco addiction treatment consists of pharmacotherapy combined with behavioral support. However, approximately half of all smokers receiving such treatment are unable to achieve even short-term abstinence, highlighting the need for novel strategies to enhance cessation outcomes^1-4^.

Cue-Restricted Smoking (CRS) is a behavioral technique introduced in 2015 as a potential aid in smoking cessation^5^. In this approach, smokers undergoing pharmacological treatment are instructed to apply CRS whenever they experience the urge to smoke. The method requires the individual to interrupt any ongoing activity, adopt a standing position, and smoke while facing a wall in an isolated location devoid of visual, auditory, or gustatory stimuli regardless of where they are or what they are doing. The rationale behind CRS is that the inconvenience of having to isolate oneself from smoking creates a negative association with the act of smoking, thereby reinforcing abstinence.

In an initial observational study, CRS was combined with varenicline, resulting in a significantly higher cessation rate at 12 weeks compared to the conventional behavioral approach (77% vs 54%, p<0.001)^5^. Based on these promising results, CRS has been adopted as the standard behavioral intervention in our Smoking Cessation Program at the Heart Institute, University of São Paulo, Brazil. CRS was also employed as the control behavioral strategy in a randomized controlled trial (RCT) evaluating pharmacogenetic guided therapy versus varenicline^6^. In that trial, 74% of participants in the control group who received CRS with varenicline achieved continuous abstinence from weeks 8 to 12, mirroring outcomes from the earlier observational study^5^.

In the context of a separate RCT investigating the efficacy of deep transcranial magnetic stimulation (dTMS) for smoking cessation, we conducted an observational sub-analysis to assess the potential impact of CRS. This was particularly relevant given the trial’s negative findings that cessation rates did not differ significantly between the dTMS and sham groups^7^. CRS was applied as part of the behavioral intervention in both active and control groups, among the participants who remained in the trial after the second week of treatment. Therefore, this sub-analysis focuses on evaluating the potential independent contribution of CRS initiated after week 2 in smoking cessation. Notably, no pharmacological therapies were provided in either group during this phase.

## METHODS

### Study design

We conducted a randomized, double-blind, placebo-controlled, single-center clinical trial to evaluate the efficacy and safety of dTMS as an aid for smoking cessation. The study took place in Sao Paulo, Brazil, with active recruitment from July 2017 to December 2021. Participants were recruited from the general population. The trial was approved by the Institutional Ethics Committee of the University of Sao Paulo (CAAE: 35068014.4.0000.0068; Heart Institute record number: 052/16/021) and registered at ClinicalTrials.gov (NCT03264313). All participants provided written informed consent before engaging in any study-related activities. Inclusion criteria were adults aged 18–70 years, smoking ≥10 cigarettes/day. Exclusion criteria were major psychiatric disorders, cognitive impairment, and use of pharmacological treatments for cessation. A total of 100 smokers were randomized: 50 to the active dTMS group and 50 to the Sham stimulation group^7^. The dTMS protocol consisted of a 20-minute stimulation session administered daily (Monday to Friday) for the first 3 weeks, followed by weekly sessions until week 6, and then biweekly sessions through week 12. Participants were instructed to stop smoking by the end of week 1. Smoking abstinence was confirmed by exhaled carbon monoxide (COex) ≤3 ppm and plasma cotinine levels <25 ng/mL^8-10.^

At each session, tobacco consumption was recorded and COex levels were measured to validate abstinence. Plasma cotinine was measured at weeks 1 and 12. Nicotine dependence was assessed using the Fagerström test for nicotine dependence (FTND)^11^, while withdrawal symptoms were measured using the Minnesota Nicotine Withdrawal Scale (MNWS)^12^ and the Tobacco Craving Questionnaire (TCQ)^13-15^. MNWS and TCQ were administered before the first stimulation session, at the end of weeks 2 and 3, and at every subsequent visit.

**Figure 1:**
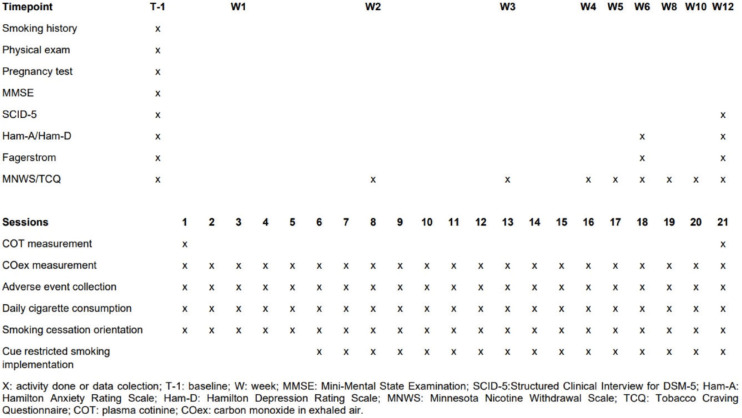
Schedule of activities of dTMS randomized control trial

Neuropsychological screening was performed prior to inclusion using the Structured Clinical Interview for DSM-5 (SCID-5)^16^, Hamilton Depression Rating Scale (HAM-D)^17^, Hamilton Anxiety Rating Scale (HAM-A)^18^, and the Mini-Mental State Examination (MMSE)^19^. The full treatment schedule is presented in Figure 1.

As the main study has already been published and demonstrated no efficacy of dTMS for smoking cessation, we performed a *post hoc* analysis to assess the impact of CRS.

### Cue-Restricted Smoking – observational sub-study

CRS was introduced in week 2 of the trial as a behavioral intervention for participants who remained in the study but had not achieved smoking cessation by the end of week 1. Fifteen participants dropped out before the second week; thus, 85 smokers continued in the protocol and received standardized instructions on how to implement CRS whenever experiencing a craving.

To assess the potential effectiveness of CRS, cigarette consumption was evaluated before and after CRS implementation. Participants were categorized into three outcome groups based on tobacco use patterns: no reduction, additional reduction, and cessation. The reduction group was further stratified by level of reduction as follows: mild (≤10%), moderate (11–49%), and significant (50–99%).

### Statistical analysis

All statistical analyses were conducted according to participants’ original randomization (active dTMS or Sham). Descriptive statistics were presented as means and standard deviations or medians and interquartile ranges for continuous variables, and as frequencies and percentages for categorical variables.

Associations between categorical variables were tested using the chi-squared test or Fisher’s exact test. Comparisons of means between two groups were performed using the Student’s t-test, and among more than two groups using one-way ANOVA. Normality of data was assessed using the Kolmogorov-Smirnov test. In cases of non-normal distributions, non-parametric alternatives (Mann-Whitney U test or Kruskal-Wallis test) were applied. *Post hoc* comparisons were performed using Duncan’s test (parametric) or Dunn-Bonferroni (non-parametric) to maintain the overall significance level. Paired comparisons over time were analyzed using paired t-tests or the Wilcoxon signed-rank test, depending on data distribution.

Changes in daily cigarette consumption and COex over time were also evaluated using Joinpoint regression models to assess temporal trends. This analysis identifies significant changes in trend (inflection points) and estimates the session percent change (SPC) for each segment, as well as the average session percent change (ASPC) for the entire series when multiple segments are present. All statistical tests were two-sided, and a significant level of 5% was adopted. Analyses were performed using SPSS version 20.0, Joinpoint Regression Program, and STATA version 17.

## RESULTS

Of the 100 participants who started the main trial, 15 dropped out before week 2, while 85 remained in the trial after week 2, and were included in this *post hoc* analysis of the impact of CRS in smoking cessation. Table 1 presents a comparison between the full randomized sample (n=100), the observational CRS cohort (n=85), and the 15 participants who dropped out. No statistically significant differences were observed among the three groups in terms of demographic data, smoking history, nicotine dependence levels, or psychiatric comorbidities. Among the 85 participants, the majority were male (68%), with a mean age of 48 years. On average, they reported 31 years of smoking history, consuming 18 cigarettes per day, had a mean baseline COex of 11.4 ppm, and a median plasma cotinine level of 200 ng/mL.

**Table 1:** Baseline characteristics of participants in the full randomized sample dTMS, early dropouts (Week 1), and the Cue-Restricted Smoking cohort

*Characteristics*	*Full dTMS sample* *(N=100)*	*Early dropouts* *(N=15)*	*Cue-Restricted* *Smoking cohort* *(N=85)*	*p*
**Gender**, n (%)				0.261^a^
Female	34 (34.0)	7 (46.7)	27 (31.8)	
Male	66 (66.0)	8 (53.3)	58 (68.2)	
**Age** (years), mean ± SD	48.4 ± 11.7	47.5 ± 12.3	48.6 ± 11.6	0.733^b^
**Age at smoking initiation** (years), median (IQR)	16.0 (15.0–18.0)	15.0 (14.0–18.0)	16.0 (15.0–18.0)	0.409^c^
**Total years smoking**, mean ± SD	31.1 ± 11.5	31.9 ± 13.8	30.9 ± 11.2	0.776^b^
**Number of cigarettes at baseline**, median (IQR)	18.0 (15.0–20.0)	20.0 (15.0–20.0)	18.0 (12.5–21.0)	0.346^c^
Stable mood disorder*, n (%)	9 (9.0)	2 (13.3)	7 (8.2)	0.621^d^
**COex at baseline (ppm)**, mean ± SD	11.2 ± 5.0	10.1 ± 3.2	11.4 ± 5.3	0.335^b^
**COT at baseline** (ng/mL), median (IQR)	200.0 (188.0 to >200.0)	200.0 (177.0 to >200.0)	200.0 (188.8 to >200.0)	0.749^c^
**MNWS at baseline** (score), mean ± SD	7.8 ± 7.3	11.0 ± 8.8	7.2 ± 6.9	0.064^b^
**TCQ at baseline** (score), mean ± SD	32.0 ± 17.3	30.7 ± 19.8	32.2 ± 17.0	0.748^b^
**Ham-A** (score), mean ± SD	5.4 ± 4.1	6.1 ± 4.6	5.3 ± 4.0	0.480^b^
**Ham-D** (score), median (IQR)	4.0 (1.0–7.0)	4.0 (2.0–8.0)	3.5 (1.0–7.0)	0.526^c^
**Treatment group**, n (%)				0.401^a^
Sham dTMS	50 (50.0)	6 (40.0)	44 (51.8)	
Active dTMS	50 (50.0)	9 (60.0)	41 (48.2)	

COex: carbon monoxide in exhaled air. COT: plasma cotinine. MNWS: Minnesota Nicotine Withdrawal Scale. TCQ: Tobacco Craving Questionnaire. Ham-A: Hamilton Anxiety Rating Scale. Ham-D: Hamilton Depression Rating Scale. dTMS: deep transcranial magnetic stimulation. IQR: interquartile range.

* Stable mood disorder: anxiety and/or depression.

a Chi-square test.

b Student’s t-test.

c Mann-Whitney test.

d Fisher’s exact test.

By the end of week 1 before CRS guidance, 33% (28/85) (95% CI: 23.1–44.0) of participants showed no reduction in cigarette consumption, while 8% (7/85) (95% CI: 3.4–16.0) reported mild, 43% (37/85) (95% CI: 33–55) moderate, and 15% (13/85) (95% CI: 8–25) significant reduction. None had achieved complete cessation at that point. Table 2 displays changes in cigarette consumption following CRS implementation during the remaining study period (up to week 12). Among those with no initial reduction in week one, 43% (12/28) (95% CI: 24–61) subsequently reduced their consumption, and one participant achieved full cessation. Of those who had significant reduction in week one, 54% (7/13) (95% CI: 26–81) achieved complete cessation by week 12.

**Table 2:** Changes in cigarette consumption before and after introduction of the Cue-Restricted Smoking technique

	*Before week 2*	*After (week 2 onwards)*				
	*n (%)*	*No reduction*	*Any reduction or cessation*			
			*Mild*	*Moderate*	*Significant*	*Cessation*
**Total**, n (%)	85 (100)	37 (43.5)	6 (7)	16 (18)	12 (14.1)	14 (16.4)
**No reduction**	28 (32.9)	16	2	3	6	1
**Mild**	7 (8.2)	2	2	1	1	1
**Moderate**	37 (43.5)	13	2	12	5	5
**Significant**	13 (15.3)	6	0	0	0	7
**Cessation**	0 (0)					

Mild: up to 10% reduction in cigarette consumption. Moderate: 11–49% reduction in cigarette consumption. Significant: 50–99% reduction in cigarette consumption.

Table 3 summarizes clinical and demographic characteristics according to final smoking status: No Reduction, Additional Reduction, and Smoking Cessation. By the end of the study, 40% (34/85) (95% CI: 29–51) had achieved additional reduction and 16.5% (14/85) (95% CI: 9–26) achieved complete cessation. The remaining 43% (37/85) (95% CI: 33–54) were categorized as non-adherent to the CRS technique. There were no significant differences in clinical variables across groups, except for the TCQ score, which was significantly higher among those who quit smoking, indicating a greater reduction in craving within this subgroup.

**Table 3:** Participant characteristics according to cigarette consumption at follow-up at 12 weeks

*Characteristics*	*No reduction*	*Additional reduction*	*Cessation*	*p*
**Total**, n (%)	37 (43)	34 (40)	14 (16.5)	
**Gender**, n (%)				0.915^a^
Male	26 (70.3)	23 (67.6)	9 (64.3)	
**Age** (years), mean ± SD	46.7 ± 11.1	50.1 ± 11.4	49.9 ± 13.4	0.414^b^
**Age at smoking initiation** (years), median (IQR)	16.0 (14.0–18.5)	16.0 (15.0–18.0)	16.0 (14.8–18.0)	0.999^c^
**Total years smoking**, mean ± SD	28.8 ± 10.6	32.9 ± 10.8	32.0 ± 13.3	0.290^b^
**Number of daily cigarettes at baseline**, median (IQR)	18.0 (15.0–20.0)	19.0 (12.0–27.3)	14.5 (11.5–20.5)	0.054^c^
**Previous MD**, n (%)				0.583^a^
Normal	30 (81.1)	28 (82.4)	13 (92.9)	
Low	7 (18.9)	6 (17.6)	1 (7.1)	
**Current DM**, n (%)	2 (5.4)	4 (11.8)	1 (7.1)	0.762^d^
**COex at baseline** (ppm), mean ± SD	12.3 ± 5.7	11.7 ± 5.2	8.5 ± 3.5	0.067^b^
**COT at baseline** (ng/mL), median (IQR)	200.0 (184.3–201.0)	200.0 (200.0–201.0)	200.0 (141.5–201.0)	0.421^c^
**MNWS at baseline** (score), mean ± SD	8.1 ± 7.4	6.3 ± 6.9	7.1 ± 5.9	0.573^b^
**TCQ at baseline** (score), mean ± SD	32.9 ± 17.0	32.5 ± 16.9	29.6 ± 18.1	0.821^b^
**MNWS absolute variation** (score), median (IQR)	0.0 (0.0–1.0)	0.0 (-0.5–2.0)	1.0 (0.0–3.8)	0.419^c^
**TCQ absolute variation** (score), mean ± SD	-11.3 ± 11.7	-17.3 ± 18.0	-23.0 ± 17.8	0.066^b^
**MNWS relative variation** (%), median (IQR)	0.0 (0.0–14.7)	0.0 (-25.0–19.2)	11.3 (0.0–32.4)	0.400^c^
**TCQ relative variation** (%), mean ± SD	-41.0 ± 38.6	-37.1 ± 77.1	-80.5 ± 29.2	0.036^b^
**Ham-A at baseline** (score), mean ± SD	5.0 ± 3.9	5.4 ± 4.0	6.1 ± 4.4	0.705^b^
**Ham-D at baseline** (score), mean ± SD	4.3 ± 3.4	4.9 ± 3.8	4.3 ± 3.6	0.761^b^
**Treatment group**, n (%)				0.822^a^
Sham dTMS	18 (48.6)	19 (55.9)	7 (50.0)	
Active dTMS	19 (51.4)	15 (44.1)	7 (50.0)	

MD: mood disorders (anxiety and/or depression). COex: carbon monoxide in exhaled air. COT: plasma cotinine. MNWS: Minnesota Nicotine Withdrawal Scale. TCQ: Tobacco Craving Questionnaire. Ham-A: Hamilton Anxiety Rating Scale. Ham-D: Hamilton Depression Rating Scale. dTMS: deep transcranial magnetic stimulation. IQR: interquartile range.

a Chi-square test.

b ANOVA.

c Kruskal-Wallis test.

d Fisher’s exact test.

Table 4 presents a logistic regression analysis examining predictors of smoking cessation, including demographic variables, baseline cigarette consumption, years of smoking, COex, plasma cotinine, and group assignment (active vs Sham dTMS). None of these variables independently predicted cessation.

**Table 4:** Predictors of smoking cessation: univariate and multivariate logistic regression models (N=85)

*Variables*	*Univariate Model* *OR (95% CI)*	*p*	*Multivariate Model* *AOR (95% CI)*	*p*
**Male**	0.83 (0.35–1.97)	0.680	0.90 (0.32–2.55)	0.849
**Age** (years)	1.02 (0.99–1.06)	0.217	-	-
**Age at smoking initiation**, (years)	0.98 (0.90–1.06)	0.589	0.97 (0.84–1.11)	0.644
**Number of daily cigarettes at baseline**	0.98 (0.93–1.03)	0.415	0.98 (0.90–1.07)	0.678
**COex at baseline** (ppm)	0.93 (0.86–1.00)	0.062	0.93 (0.82–1.05)	0.216
**Total years smoking**	1.03 (0.99–1.07)	0.163	1.04 (0.99–1.08)	0.101
**COT at baseline** (ng/mL)	1.00 (0.99–1.01)	0.806	1.01 (0.99–1.02)	0.345
**Ham-D at baseline** (score)	1.02 (0.91–1.14)	0.752	0.89 (0.72–1.10)	0.293
**Ham-A at baseline** (score)	1.04 (0.94–1.16)	0.418	1.08 (0.90–1.30)	0.424
**Treatment group (dTMS/Sham)**	0.87 (0.39–1.94)	0.737	0.65 (0.26–1.66)	0.371

AOR: adjusted odds ratio. COex: carbon monoxide in exhaled air. COT: plasma cotinine. Ham-A: Hamilton Anxiety Rating Scale; Ham-D: Hamilton Depression Rating Scale. dTMS: deep transcranial magnetic stimulation.

**Figure 2:**
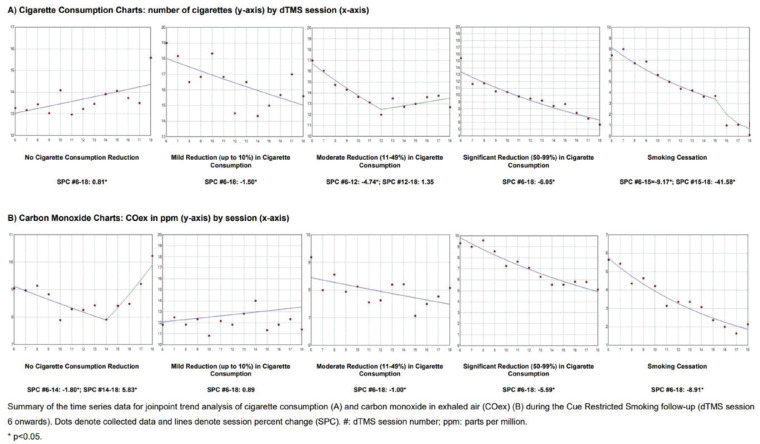
Change in cigarette consumption and COex analyzed by the Joinpoint trend test

Figure 2 shows Joinpoint regression models evaluating trends in cigarette consumption and COex. The no reduction group showed a modest but significant increasing trend in cigarette consumption (variation=0.81; 95% CI: 0.21–1.42; p=0.013). The mild reduction group demonstrated a significant decreasing trend (-1.50; 95% CI: -2.72 – -0.26; p=0.022). The moderate reduction group exhibited a robust initial decline (-4.74; 95% CI: -6.01 – -3.45; p<0.001), followed by a non-significant increase. The significant reduction group maintained a strong decreasing trend (-6.05; 95% CI: -7.19 – -4.89; p<0.001). The smoking cessation group showed a sharp drop between weeks 2 and 3 (-9.17; 95% CI: -11.17 – -7.13; p<0.001), followed by a more pronounced reduction from weeks 3 to 12 (-41.58; 95% CI: -58.17 – -18.42; p=0.006).

For COex, the no reduction group showed an initial significant decrease (-1.80; 95% CI: -3.23 – -0.34; p=0.022) followed by a rebound (5.83; 95% CI: 0.77–11.15; p=0.029). The mild reduction group showed a non-significant increasing trend. The moderate reduction group demonstrated a steady decrease (-1.00; 95% CI: -1.91 – -0.08; p=0.036), and the significant reduction group showed a clear downward trend (-5.59; 95% CI: -6.76 – -4.41; p<0.001). The smoking cessation group exhibited a strong and continuous decrease from week 2 through 12 (-8.91; 95% CI: -10.59 – -7.19; p<0.001).

## DISCUSSION

This study demonstrated that the application of CRS contributed meaningfully to cigarette reduction and cessation among smokers enrolled in a dTMS clinical trial. While many participants are usually already motivated to reduce consumption during the first treatment week^20^, CRS appeared to facilitate further reductions thereafter, with 40% of the cohort reporting additional reduction and 16.5% achieving complete abstinence (Table 2). These outcomes reinforce the feasibility and potential utility of CRS as a behavioral adjunct, consistent with previous research^5^.

Behavioral support is central to smoking cessation. Cognitive Behavioral Therapy (CBT)^21^, Motivational Interviewing^22^, Contingency Management^23^, and Mindfulness-based interventions^24^ have all shown efficacy, but often require trained personnel and structured setting resources that may be limited in low- and middle-income countries.

Importantly, those who achieved cessation had lower baseline cigarette use (Table 3), supporting evidence that low consumption smokers may quit successfully with behavioral support alone^25^. The quit rate observed here (16.5%), confirmed via biochemical markers, is higher than typical placebo-controlled pharmacotherapy trials (about 10%)^2^.

Longitudinal data analysis (Figure 2) revealed that CRS-induced reductions occurred progressively. TCQ scores declined in the cessation group without marked increases in withdrawal symptoms in the reduction group, suggesting favorable neuroadaptation. These findings align with mechanisms involving receptor downregulation during sustained abstinence^26,27^.

### Limitations

Limitations of this study include its observational design and the fact that data were derived from a secondary analysis. While we adjusted for potential confounders, residual confounding cannot be excluded. Smoking behavior was partially assessed by self-report, which introduces the potential for information bias, although objective biochemical confirmation was also used. The absence of a structured control group for CRS limits causal inference. Moreover, the generalizability of findings may be constrained by the single-center nature of the study conducted in Sao Paulo, Brazil. Nevertheless, this analysis provides initial evidence supporting CRS as a low-cost, low-complexity behavioral technique that warrants further investigation.

## CONCLUSIONS

Given its simplicity, absence of cost, and ease of implementation, Cue-Restricted Smoking represents a promising behavioral strategy that can be readily integrated into smoking cessation programs, particularly in low-resource settings where access to pharmacological or specialized behavioral therapies may be limited. Future randomized controlled trials are needed to validate these findings and explore broader applications across different cultural contexts.

## Data Availability

The data supporting this research are available from the authors on reasonable request.
